# Improved eating behaviours mediate weight gain prevention of young adults: moderation and mediation results of a randomised controlled trial of TXT2BFiT, mHealth program

**DOI:** 10.1186/s12966-016-0368-8

**Published:** 2016-04-02

**Authors:** Stephanie R. Partridge, Kevin McGeechan, Adrian Bauman, Philayrath Phongsavan, Margaret Allman-Farinelli

**Affiliations:** School of Life and Environmental Sciences, Charles Perkins Centre, University of Sydney, Sydney, NSW 2006 Australia; Sydney School of Public Health, Charles Perkins Centre, University of Sydney, Sydney, NSW 2006 Australia

**Keywords:** Young adults, Obesity prevention, Nutrition, Lifestyle, mHealth, Mediation, Moderation

## Abstract

**Background:**

Explanatory evaluation of interventions for prevention of weight gain is required beyond changes in weight, to determine for whom the intervention works and the underlying mechanisms of change. It was hypothesised that participant characteristics moderate intervention effect on weight change and improved eating and physical activity behaviours during the 3-month program mediate the relationship between intervention and weight.

**Methods:**

In our randomised controlled trial, young adults at risk of weight gain (*n* = 250) were assigned either to an intervention group that received a 3-month mHealth (TXT2BFiT) program with 6-month maintenance or to a control group. Data were collected via online self-report surveys. Hypothesised moderators and mediators of the intervention effect on weight were independently assessed in PROCESS macro models for 3 and 9-month weight change.

**Results:**

Males (*P* = 0.01), mid-20s age group (*P* = 0.04), and higher income earners (*P* = 0.02) moderated intervention effects on weight change at 3-months and males only at 9-months (*P* = 0.02). Weight change at 3 (−1.12 kg) and 9-months (−1.38 kg) remained significant when 3-month nutrition and physical activity behaviours were specified as mediators (*P* <0.01 and *P* = 0.01 respectively). Indirect paths explained 39 % (0.72/1.85 kg) and 40 % (0.92/2.3 kg) of total effect on weight change at 3 and 9-months respectively. Increased vegetable intake by intervention group at 3-months accounted for 19 and 17 % and decreased sugar-sweetened beverages accounted for 8 and 13 % of indirect weight change effects at 3 and 9-months respectively.

**Conclusions:**

TXT2BFiT was effective for both young men and women. Small sustained behavioural changes, including increased vegetable intake and decreased sugar-sweetened beverages consumption significantly mediated the intervention’s effects on weight change. Improved eating behaviours and increased physical activity accounted for approximately 40 % of the weight change.

**Trial registration:**

The trial is registered with the Australian New Zealand Clinical Trials Registry (ACTRN12612000924853).

## Background

Obesity is a major global challenge due to the substantial increases in prevalence and associated health risks [1]. Australia is no exception and young adults are the group gaining weight the most rapidly [2], and at a faster rate than in previous generations [3]. In 2014–15, 38.9 % 18 to 24 year olds and 52.4 % of 25 to 34 year olds in Australia were overweight or obese [4].

It is recognised that improvements in eating and physical activity behaviours have major health benefits for later life including the prevention of weight gain e.g. increase in fruit and vegetable intake [5], and maintaining high activity levels through young adulthood may lessen weight gain as young adults transition to middle age [6].

Lifestyle interventions for weight gain prevention in young adults have demonstrated effectiveness [7]. Developing engaging and technology focused interventions may be useful to maintain healthy lifestyle behaviours for young adults. Young adults have deeply embedded mobile devices into their lives, with smartphone ownership and use highest in Australian and American 18 to 35 year olds (95 and 92 % respectively) [8]. There remains insufficient evidence for effective eHealth and mHealth weight gain prevention interventions [9]. There are emerging interventions from Australia and America for use of mHealth technology for weight gain prevention in young adults [10].

To improve effectiveness of prevention interventions, it is important to investigate for whom (moderators analysis), and how (mediators analysis) interventions worked - that is, the mechanisms underlying behavioural change. Moderation and mediation analysis are methods commonly used to answer such questions [11, 12]. Moderation analysis is important to assess the effect of the intervention on different subgroups of participants and staff delivering programs to assess differing delivery effects. Mediation analysis is important to investigate potential associations, such as increases in healthy eating and physical activity resulting in change in weight.

Moderation and mediation analysis are important components for generalisability and future program implementation [12]. Improved eating and physical activity behaviours have been shown to already mediate prevention of weight gain in middle-aged women [13] and mediate weight loss in overweight and obese adults [14, 15]. Our recent review of external validity reporting in weight gain prevention interventions for young adults identified limited investigation of moderation or mediation analysis in studies of young adults [16]. Such information is needed in order to further develop interventions.

The ‘TXT2BFiT’ mHealth intervention was efficacious for weight gain prevention [17]. The program aimed to counsel participants at risk of weight gain to improve their eating and physical activity behaviours, with an overall aim of maintaining or reducing weight [18]. In addition to weight loss, eating behaviours and physical activity demonstrated favourable change [17]. This paper aimed to examine whether prevention of weight gain was associated with individual factors. The second aim was to examine the hypothesised mediating effects of healthy eating and physical activity on weight change.

## Methods

### Study design

The randomised controlled trial duration was 3-months followed by an additional 6-month maintenance phase. The trial was approved by the University Human Research Ethics Committee in September 2012 (Approval Number 15226) and all the participants gave written informed consent. The trial was registered with the Australian New Zealand Clinical Trials Registry (ACTRN12612000924853).

### Participant recruitment and inclusion criteria

Two-hundred and fifty young adults at risk of weight gain were recruited between November 2012 and July 2014 from the Greater Sydney Area, Australia via primary care and print and electronic media. Detailed recruitment information is available elsewhere [19]. Eligible individuals were 18 to 35 years old and at risk of weight gain (BMI between 23.0 and 24.9 kg/m^2^ and a reported 2 kg weight gain in the previous 12-months) or were overweight or obese with a BMI between 25.0 and 31.9 kg/m^2^. Although the BMI cut point for overweight is 25.0 kg/m^2^ [20], individuals with a BMI as low as 23.0 kg/m^2^ with self-reported weight gain were considered acceptable for this program to halt further weight gain. A cut point of 32.0 kg/m^2^ was considered acceptable for the programme, whereas, above this level, more intensive intervention for weight loss would be indicated. Individuals were required not to meet the daily recommended fruit and/or vegetable intake per day, had a sugar sweetened beverage (SSB) intake in excess of 1 litre weekly; had energy-dense meals prepared away from home (i.e. take-out food) more than once per week, and/or engaged in moderate-intensity physical activity of less than 60 min daily. Individuals were excluded if they were pregnant or planning to fall pregnant within the study period, were enrolled in an alternate weight loss program, had lost greater than 10 kg in the past 3 months, taken medications that have caused weight gain of greater than 2 kilograms, had medical conditions that precluded following dietary or physical activity recommendations, and/or did not speak English. Participants were also required to have a mobile phone capable of receiving text messages and accessing the internet at least once a week. Participants were blinded to the allocation until after completion of the 9-month study.

### Intervention group

Detailed information of the TXT2BFiT program is available elsewhere [17, 18]. In brief, each intervention participant received the 3-month TXT2BFiT program consisting of five personalised coaching calls with a dietitian, eight weekly gender and stage-of-change specific text messages targeting fruit and vegetable consumption, take-out meal consumption, SSB consumption and physical activity levels, weekly emails and access to smartphone applications and study website. The maintenance phase lasted 6-months and participants received two booster coaching calls, monthly text messages and emails and had ongoing access to the smartphone applications and website. Two female interventionists (dietitians) in the target age group delivered the TXT2BFiT program.

### Control group

The control group received a two page handout based on the Australian Dietary Guidelines and National Physical Activity Guidelines [21, 22], an introductory phone call (no coaching given) and four text messages over the first 3-months with no additional intervention.

### Measures

Data collection took place at baseline, 3- and 9-months. Measures were conducted via online surveys in which all participants were asked their age, gender, postcode (for categorizing socio-economic status (SES) [23]), ethnicity (language spoken at home) [24], income bracket [24], education level [24], recruitment source, relationship status and living arrangement. The primary outcome was self-reported body weight (kg). Validated short questions were used to categorise daily intake of fruits [25], vegetables [25], usual weekly intake of SSB [25] and weekly takeout meals [26]. The short dietary questions were on a sliding scale, with a higher score, indicating a more desired response and for the purpose of the mediation analysis were used as continuous variables, referred to as food scores. Fruit and vegetables were scored one (zero serves per day) through seven (six or more serves per day) with a difference of one unit representing approximately one serve per day. SSB was scored one (zero or diet per week) through five (3000 mL or more per week) with a difference of one unit representing one litre (L) per week. Take-out meals was scored one (one or less per week) through four (six to seven per week) with a difference of one unit representing one to two take-out meal per week.

Questions about physical activity in the previous 7 days was measured using the International Physical Activity Questionnaire short form (IPAQ-SF) [27]. The IPAQ-SF was scored using established methods and data were reported as a continuous measure in metabolic equivalent of task (MET)-minutes per week. Days of walking, moderate and vigorous physical activity were totalled per week from the IPAQ-SF questionnaire to determine total physical activity days continuous measure [28]. Trained researchers analysing the results were blinded to participant allocation.

### Statistical analysis

The moderation and mediation analysis was conducted in SPSS Statistics Version 22 (SPSS Inc, Chicago, Illinois, USA) investigating whether weight loss at 3- and 9-months were moderated and/or mediated by participant characteristics and change in eating and physical activity behaviours. Missing values were imputed using the expectation maximisation imputation technique in SPSS for the primary outcomes, body weight (kg) and for the secondary outcomes, food scores, physical activity MET minutes and physical activity days in the mediation analysis. To adjust for pre-intervention effects, baseline values, allocation, general practitioner practice and gender were included as covariates in the moderation and mediation models.

### Moderation analysis

All baseline participant characteristics hypothesised to moderate the effect of the intervention on body weight were independently assessed in single moderation models for 3- and 9-month weight change. The PROCESS SPSS Macro version 2.13, models 1 and 2 [29] were used to calculate the regression coefficients for each participant characteristic independently. The procedure of Hayes for moderation analysis was used for binary covariates (gender, SES, ethnicity, income, significant other, living situation and interventionist) [29]. For participant characteristics with three or more categories, they were collapsed to three to be included in the moderation model as outlined in the procedure in Hayes, [30].

### Mediation analysis

**Fig. 1:**
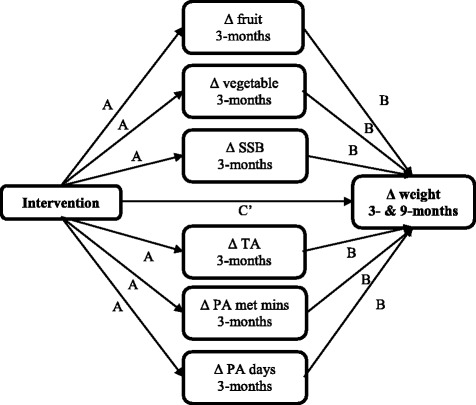
Mediation pathway for food and physical activity behaviours hypothesised to mediate weight gain prevention for intervention participants in the TXT2BFiT study. A = unstandardised regression coefficient of the intervention allocation predicting hypothesised mediators; B = unstandardised regression coefficient of the hypothesised mediator predicting weight with intervention allocation included in the model and C’ = unstandardised regression coefficient of the intervention allocation predicting change in weight with mediator in the model. *SSB* sugar sweetened-beverages, *TA* take-out meals, *PA* physical activity, *MET* mins, metabolic equivalent of task minutes

Each of the six continuous food scores (fruits, vegetables, SSB and take-out meals) and physical activity measures (MET minutes and total physical activity days) at 3-months hypothesised to mediate the effect of body weight change was assessed in a multiple mediation model for 3- and 9-month body weight change (Fig. 1). The PROCESS SPSS Macro version 2.13, model four [31] was used to calculate four pathways. Pathway A determined the regression coefficients for the effect of the intervention on 3-month dietary scores and physical activity measures, Pathway B examined the association between changes in 3-month dietary scores and physical activity measures and changes in body weight at 3- and 9-months, independent of allocation, and Pathway C and C’ estimated the total and direct effect of the intervention on 3- and 9-month body weight change respectively. Pathway AB calculated the indirect intervention effects. To test the significance of the indirect effect, the macro generated bias-corrected bootstrapped 95 % confidence intervals (CI) [31]. Significant mediation was established if the CI around the indirect effect did not include zero [31]. The moderation and mediation analysis were repeated for body mass index (BMI) as the primary outcome variable.

## Results

Two-hundred and fifty participants were randomly allocated to the intervention group (*n* = 125) or control group (*n* = 125) in a one-to-one ratio. Online baseline surveys were completed by 248 participants (intervention, *n* = 123 and control, *n* = 125). Baseline demographics were similar between intervention and control groups. See Partridge et al., [17] for more information regarding baseline demographics, a participant flow diagram, loss/exclusions after randomisation and adherence.

### Moderation analysis

Participant characteristics hypothesised to moderate change in body weight are shown in Table 1. At 3-months, age category (*P* = 0.04) significantly moderated the effect of the intervention on weight change but not at 9-months (*P* = 0.65). Participants aged 18–24 years weighed 1.1 kg (95 % CI −2.3, 0.6) less compared to controls (*P* = 0.06) at 3-months. Participants aged 25–29 and 30–35 years weighed significantly less weight compared to controls (−3.2 kg 95 % CI −4.8, −2.0, *P* <0.01 and −1.5 kg 95 % CI −2.5, −0.5, *P* <0.01 respectively) at 3-months. At both 3- and 9-months males lost more weight than females, however, both genders achieved significantly greater weight loss than their control counterparts. Participants earning a higher income weighed less than those earning a lower income at 3-months (*P* = 0.02), however, both income groups achieved significantly greater weight loss than their control counterparts. No other participant characteristics or interventionists moderated weight change at 3- or 9-months. Results for BMI change were consistent with results for weight change.

**Table 1:** Participants’ baseline characteristics identified as potential moderators and moderated intervention effects on weight change at 3- and 9-months

Baseline characteristic	Control (*n* = 125)		Intervention (*n* = 123)^a^		Moderated effect on weight at 3-months			Moderated effect on weight at 9-months		
	*n*	*%*	*n*	*%*	*Diff*	*SE*	*P*	*Diff*	*SE*	*P*
Age category										
18–24 years	38	30.4	36	29.3	−1.1	0.6	0.04	−1.6	1.0	0.65
25–29 years	39	31.2	27	22.0	−3.2	0.7		−2.8	1.0	
30–35 years	48	38.4	60	48.8	−1.5	0.5		−2.5	0.8	
Gender										
Female	79	63.2	73	59.3	−1.1	0.4	0.01	−1.3	0.7	0.02
Male	46	36.8	50	40.7	−2.8	0.5		−3.8	0.8	
Socioeconomic status										
1st, 2nd & 3rd quintiles^b^	7	5.6	8	6.5	−0.7	1.4	0.40	−3.5	2.1	0.06
4th & 5th quintiles^c^	118	94.4	115	93.5	−1.9	0.3		−2.3	0.5	
Ethnicity										
English	90	72.0	82	66.7	−2.2	0.4	0.08	−2.3	0.6	0.79
Other	35	28.0	41	33.3	−0.9	0.6		−2.0	0.9	
Education level										
High school or below	21	16.8	27	22.0	−0.8	0.8	0.35	−1.6	1.2	0.81
Some university or technical school	25	20.0	22	17.9	−1.9	0.8		−2.2	1.2	
University bachelor degree or higher	79	63.2	74	60.2	−2.1	0.4		−2.5	0.7	
Income (AU$)										
<$AUS 80,000	94	75.2	100	81.3	−1.4	0.4	0.02	−1.9	0.6	0.20
>$AUS 80,000	31	24.8	23	18.7	−3.2	0.7		−3.5	1.1	
Recruitment source^d^										
GP letter	31	25.4	37	31.1	−1.3	0.6	0.64	−1.7	1.0	0.56
Print Media	39	32.0	30	25.2	−1.6	0.6		−3	1.0	
Electronic media	52	42.6	52	43.7	−2	0.5		−1.8	0.8	
Significant other										
Yes	68	54.4	76	61.8	−2.2	0.4	0.14	−2.5	0.7	0.79
No	57	45.6	47	38.2	−1.2	0.5		−2.2	0.8	
Living situation										
Alone	16	12.8	12	9.8	−2.5	1.0	0.47	−2.4	1.6	0.92
Not alone	109	87.2	111	90.2	−1.7	0.4		−2.3	0.6	
Interventionist										
Dietitian 1	74	59.2	77	61.6	−2.1	0.4	0.31	−2.6	0.7	0.51
Dietitian 2	51	40.8	48	38.)	−1.4	0.5		−1.9	0.8	

*AU$* Australian Dollars, *Diff* difference, *GP* general practitioner, *SE* standard error; ^a^All participants had measured variables excluding two participants who did not complete baseline self-report surveys; ^b^Lowest quintiles; ^c^Highest quintiles; ^d^Seven participants (three control, six intervention) did not recall their recruitment source and were excluded from the moderation analysis on recruitment source

### Changes in body weight, food and physical activity behaviours during the intervention

Means for body weight, food scores and physical activity measures over the course of the 9-month intervention are shown in Table 2. At the end of 3-months, the estimated difference in weight change between the intervention and control group was −1.8 kg (95 % CI −2.5, −1.2, *P* <0.01). At the end of 9-months, the estimated difference in weight change between the intervention and control group was −2.3 kg (95 % CI −3.3, −1.3, *P* <0.01). At the end of 3-months, the estimated difference in fruit score (0.20, 95 % CI 0.03, 0.38, *P* = 0.025), vegetable score (0.46, 95 % CI 0.22, 0.65, *P* <0.01), SSB score (0.49, 95 % CI 0.23, 0.75, *P* <0.01) and take-out meal score (0.25, 95 % CI 0.09, 0.40, *P* <0.01) were significantly different between the intervention and control group. The estimated difference in total physical activity days per week between the intervention and control group was 1.1 days (95 % CI 0.32, 1.86, *P* <0.01). The estimated difference in total physical activity MET minutes per week did not differ significantly between the intervention and control group (251.9 MET minutes, 95 % CI −138.8, 642.6, *P* = 0.21). At the end of 9-months, the estimated difference in food scores remained significantly different between the intervention and control group. The estimated difference in total physical activity MET minutes and total physical activity days per week did not differ significantly between the intervention and control group. The changes at 3-months were specified in the mediation model for 3- and 9-month weight change.

**Table 2:** Means and standard deviations for weight, food scores^a^ and physical activity measures by allocation at baseline, 3- and 9-months and time specific difference at 3-months after controlling for allocation, practice, gender and baseline values

Outcomes	Baseline		3-months		9-months	
	*Mean*	*SD*	*Mean*	*SD*	*Mean*	*SD*
Weight, kg						
Control	79.3	12.6	78.8	12.6	78.4	12.8
Intervention	78.4	11.2	76.0	10.7	74.9	10.8
*Time specific difference I-C (95 % CI)*			−1.8 (−2.5, −1.2)	*P* <0.001	−2.3 (−3.3, −1.3)	*P* <0.001
Fruit score^b^						
Control	2.54	0.93	2.80	0.89	2.63	0.88
Intervention	2.40	0.70	2.95	0.81	3.01	0.85
*Time specific difference I-C (95 % CI)*			0.20 (0.03, 0.38)	*P* = 0.025	0.36 (0.17, 0.54)	*P* <0.001
Vegetable score^b^						
Control	3.28	1.13	3.64	1.22	3.63	1.22
Intervention	3.28	1.22	4.11	1.24	4.13	1.22
*Time specific difference I-C (95 % CI)*			0.46 (0.22, 0.65)	*P* <0.001	0.41 (0.18, 0.65)	*P* = 0.001
SSB score^c^						
Control	4.12	0.97	4.29	0.83	4.34	0.72
Intervention	4.17	0.91	4.57	0.52	4.58	0.52
*Time specific difference I-C (95 % CI)*			0.49 (0.23, 0.75)	*P* <0.001	0.27 (0.12, 0.41)	*P* <0.001
Take-out meal score^d^						
Control	3.12	0.83	3.36	0.82	3.45	0.77
Intervention	3.20	0.80	3.71	0.51	3.68	0.61
*Time specific difference I-C (95 % CI)*			0.25 (0.09, 0.40)	*P* = 0.002	0.17 (0.02, 0.32)	*P* = 0.032
PA, MET minutes per week						
Control	1646.78	1474.61	1861.84	1687.22	2318.30	2033.76
Intervention	1619.93	1581.14	2210.52	2255.98	2404.78	1855.67
*Time specific difference I-C (95 % CI)*			251.9 (−138.8, 642.6)	*P* = 0.21	76.98 (−354.39, 508.36)	*P* = 0.73
PA, total days of PA per week						
Control	7.36	3.83	7.77	3.79	8.49	4.19
Intervention	6.63	3.33	8.80	3.71	8.73	3.47
*Time specific difference I-C (95 % CI)*			1.1 (0.32, 1.86)	*P* = 0.005	0.48 (−0.37, 1.33)	*P* = 0.27

*CI* confidence interval, *C* control, *I* intervention, *MET* minutes, metabolic equivalent of task minutes, *SD* standard deviation, *SSB* sugar-sweetened beverages

^a^Validated short questions were on a sliding scale, with a higher score, indicating a more desired response and for the purpose of the mediation analysis were used as continuous variables, referred to as food scores; ^b^Fruit and vegetables were scored one (zero serves per day) through seven (six or more serves per day) with a difference of one unit representing approximately one serve per day; ^c^SSB was scored one (zero or diet per week) through five (3000 mL or more per week) with a difference of one unit representing one litre (L) per week; ^d^Take-out meals was scored one (one or less per week) through four (six to seven per week) with a difference of one unit representing one-two take-out meals per week

### Mediation analysis

Table 3 shows pathway A, which tested the direct effects of being in the intervention group for the potential mediators. At 3-months, significant differences between intervention and control groups were observed for all except one of the hypothesised mediators while adjusting for all other variables in the model. The associations between changes in mediators from baseline to 3-months and changes in weight at 3- and 9-months are also shown in Table 3. After controlling for baseline values, the intervention effect on the vegetable score was an increase of 0.48 units on a seven point scale (*P* <0.01), which represents an approximate increase of half a serve of vegetables per day. There was a significant inverse association between vegetable score for vegetable intake and weight change at 3- (*P* <0.01) and 9-months (*P* <0.01), demonstrating that increased vegetable intake in the 3-month program was associated with greater weight change at 3- and 9-months, regardless of allocation. The intervention effect on vegetable score significantly mediated the effect on weight at both 3- (AB = −0.34, 95 % CI −0.70, −0.11) and 9-months (AB = −0.39, 95 % CI −0.92, −0.08). The mediating effect of increasing vegetable score was found to account for 18.5 and 17.0 % of the intervention effect of weight change (C’) at 3- and 9-months respectively. The effect of the intervention on SSB score at 9-months was an increase of 0.61 units (*P* = 0.02), which represents an approximate decrease of 500 mL per week. The intervention effect on SSB score significantly mediated the effect on weight at 3- (AB = −0.14, 95 % CI −0.34, −0.04) and 9-months (AB = −0.29, 95 % CI −0.64, −0.07), accounting for 7.6 and 17.4 % of the intervention effect on weight change respectively. No other diet score of physical activity measures significantly mediated weight loss.

**Table 3:** Effect of the intervention on potential mediators and the associations between changes in mediators and changes in weight at 3- and 9-months (using imputation for missing data)

Hypothesized mediators	Month	Direct effect of intervention on weight			Intervention effect on potential mediators			Association between potential mediators and weight change			Mediated effect			
		C’	(SE)	*P*	A	(SE)	*P*	B	(SE)	*P*	AB	(SE)	95 % CI	AB/(C’ + AB)
Fruit	3	−1.12	0.34	<0.01	0.21	0.09	0.02	−0.30	0.24	0.24	−0.06	0.07	−0.27, 0.04	3.26 %
	9	−1.38	0.55	0.01				−0.10	0.39	0.82	0.00	0.10	−0.23, 0.19	0.17 %
Vegetables	3	−1.12	0.34	<0.01	0.48	0.12	<0.01	−0.70	0.18	<0.01	−0.34	0.14	−0.70, −0.11	18.48 %
	9	−1.38	0.55	0.01				−0.79	0.28	<0.01	−0.39	0.20	−0.92, −0.08	17.00 %
SSB	3	−1.12	0.34	<0.01	0.27	0.08	<0.01	−0.50	0.27	0.07	−0.14	0.09	−0.34, −0.04	7.61 %
	9	−1.38	0.55	0.01				−0.61	0.26	0.02	−0.29	0.14	−0.64, −0.07	17.37 %
Take-out meals	3	−1.12	0.34	<0.01	0.29	0.07	<0.01	−0.38	0.30	0.20	−0.11	0.10	−0.35, 0.06	5.41 %
	9	−1.38	0.55	0.01				−0.34	0.44	0.44	−0.11	0.13	−0.40, 0.11	4.80 %
PA MET mins	3	−1.12	0.34	<0.01	221.45	201.8	0.27	0.00	0.00	0.20	−0.03	0.06	−0.22, 0.03	1.63 %
	9	−1.38	0.55	0.01				0.00	0.00	0.43	0.04	0.05	−0.02, 0.21	−1.74 %
PA total days	3	−1.12	0.34	<0.01	0.95	0.39	0.02	−0.04	0.06	0.49	−0.04	0.08	−0.24, 0.10	2.17 %
	9	−1.38	0.55	0.01				−0.16	0.1	0.12	−0.16	0.14	−0.53, 0.02	6.97 %

Table design adapted from Hollis et al. (2013) [13]; C’ = unstandardised regression coefficient of the intervention predicting change in weight with mediator in the model. (*SE* standard error); A = unstandardised regression coefficient of the intervention condition predicting hypothesised mediators; B = unstandardised regression coefficient of the hypothesised mediator predicting weight with intervention condition included in the model; AB = product-of-coefficients estimate. (95 % CI = 95 % confidence interval, Bootstrap bias corrected 95 % confidence intervals of the mediated effect); AB/(C’ + AB) = Proportion of intervention weight effect that was mediated

The total effect (C) on weight change equalled the direct effect (−1.12 kg) plus the indirect effect (−0.72 kg), which equalled −1.85 kg (*P* <0.01) at 3-months. The indirect effect is composed of the combination of all diet score and physical activity effects. This indicated that the indirect paths explained 38.9 % of the total effect (−0.72/−1.85 kg). A total of 47.2 % of the indirect effect was related to vegetable score change and 19.4 % of the indirect effect is accounted for by the SSB score change. Total effect (C) on weight change equalled the direct effect (−1.38 kg) plus the indirect effect (−0.92 kg), which equalled −2.3 kg (*P* = 0.01) at 9-months. This indicated that the indirect paths explained 40.0 % of the total effect (−0.92/−2.3 kg). A total of 42.4 % of the indirect effect was related to vegetable score change and 31.5 % of the indirect effect is accounted for by the SSB score change. Mediation analysis results with BMI change as the outcome variable were consistent with weight change.

## Discussion

The results supported our hypothesis that improvements in eating and physical activity behaviours made during the 3-month intervention mediate the effect of the intervention on weight change post-intervention (3-months) and during maintenance (9-months). The mediation model revealed the mHealth program, TXT2BFiT had positive effects on vegetable and SSB intake that contributed to weight loss. This finding is consistent with previous research that indicate vegetable intake and SSB consumption are related to weight management [5, 32]. Gender was the only participant characteristic at 9-months that moderated the intervention effect on weight change. This finding is consistent with previous research that indicate males lose more weight than females [33].

The findings of this moderator analysis are relevant, given the population that enrolled in the TXT2BFiT study. More men than previous research suggests enrolled (approximately 40 %) [34], and subsequently we were able to detect that gender moderated the intervention effect on weight change. Men in the intervention group achieved greater weight loss than women in the intervention group, possibly due to the greater baseline weight of men and higher caloric intake rather than our specific lifestyle intervention [33]. Previous programs have targeted middle-aged men [35, 36], however, no weight gain prevention interventions thus far have been specifically designed for young men nor delivered using mHealth. This low intensive and flexible intervention shows promise for men. Despite men being more successful, our program was still effective for women as well, in line with current findings in weight change interventions [33]. Some of the intervention content was tailored for gender, (e.g. all text messages were gender specific and also the coaching calls were personalised) as recommended by a recent systematic review investigating smoking, nutrition, alcohol, physical activity and obesity in young men [37]. The coaching calls used a participatory approach, designed to address individual participant characteristics within the study population [38], based on our pilot research [39]. Furthermore, our process evaluation had a high young male participatory rate with no gender differences noted. This shows promise for future scale up and generalisability.

It is important to note that this analysis was exploratory and the data was not originally powered for subgroup analysis. Age was found to moderate the effect of the intervention on weight change at 3-months, with those aged 25–29 years losing the most weight. Both baseline weight and BMI in this sample increased with age and younger adults, aged 18–24 years in the intervention group, were still able to lose 1.1 kg at 3-months. The age effect diminished at 9-months with no difference between age groups. Motivating factors for weight loss or weight loss maintenance have been shown to be age dependent [40]. Differing lifestyle factors, such as marital status, occupational status, housing environment, educational attainment and family circumstances may be attributable to difference observed with those over 25 years [41]. The personalised coaching calls allowed for individual age-specific motivators and goals to be taken into account.

A unique contribution of the moderation analysis shows that the intervention was equally effective when the interventionist (dietitian) changed. It is rarely reported in prevention interventions if the interventionist is a moderating factor of the program outcome, however, it is an important external validity component [16, 42]. Our process evaluation revealed that participants, regardless of interventionist felt a sense of accountability. As such, both interventionists were successful in prompting behaviour change and subsequent weight loss in intervention participants. The intervention procedures were flexible for personalisation, however, still allowed for consistency of outcomes.

The mediation analysis results support the process-of-change theory, which suggests that self-monitoring, motivational interviewing and goal setting can help change behaviours of interest [43]. The behavioural changes made by the participants in the first 3 months are crucial for weight change at 3 months. If modelling some behaviour change, in this case decreased SSB consumption and increased vegetable intakes, can be achieved during the 3-month period then this increases likelihood of effective subsequent weight loss or preventing weight gain at intervention completion. This finding is consistent with the empirical literature indicating that 3 to 6 months as being the critical period for establishing behavioural change [44, 45]. Through its various mHealth intervention components, the TXT2BFiT program consistently encouraged participants to modify risk behaviours to prevent weight gain. The findings do suggest that the mHealth platform was an effective delivery medium for the theoretical components required to cause behaviour change [46], as found by Norman et al., [15] with a text message weight loss intervention.

Our analysis also suggests that other factors not in the mediation model may contribute to the relationship between the intervention group and weight loss. Less than half of the effect of the intervention was explained by the mediating pathways at each time point (i.e., 38.9 and 40.0 % respectively), indicating that much of the between group variation in weight change was from factors not accounted for in the model but may be related to being randomized to the intervention group. Text messages, email, smartphone applications and website resources targeted four eating behaviours the majority of young Australians do not follow. As these eating behaviours were significantly improved in the intervention group compared to the control at 3- and 9-months they were included in the mediation model. However, the dietitian delivered coaching calls allowed participants to personalise eating and physical activity goals. As shown at baseline, enrolling participants in the TXT2BFiT study had diet qualities poorer than that of a national representative sample [47]. Discretionary choices and alcohol (approximately 33.2 % of total energy intake) were consumed highly in the study sample [47], and are classified as energy-dense nutrient poor choices. Participants may have reduced consumption of discretionary choices and alcohol, which wasn’t accounted for in the mediation model. In addition, purposively designed study resources emphasised appropriate portion sizes of core foods, reducing added sugar and reducing alcohol based on the national guidelines. If adhered this would result in an energy deficit required for weight loss.

An undetected increase in energy expenditure, through structured or unstructured physical activity may have accounted for a portion of the unexplained weight loss (−1.12 kg at 12-weeks and −1.38 kg at 36-weeks). Text messages encouraged participants to decrease their sedentary time and incorporate unstructured physical activity into their day. Mediation analyses conducted in another weight gain prevention intervention for middle-aged women showed adherence to 10,000 steps per day mediated the effect intervention effect on weight change [13]. Pedometers worn by participants may have been an objective way to measure increased incidental physical activity [13], which was unable to be accounted for in the present study. Despite the convenience of implementation, the self-reported physical activity measure used in the current study (IPAQ-SF) as an indicator of relative or absolute physical activity is weak [27].

### Strengths and limitations

The analysis included a multiple mediation model to identify the unique contribution of each eating and physical activity behaviour variable to changes in weight. There are several benefits of including multiple mediators in a single model, including obtaining relative magnitudes of the specific indirect effects associated with all mediators [31]. Missing data was accounted for with an intention-to-treat analysis, however, only a single imputation method was used. This analysis was exploratory and as with most RCTs, the data was originally powered for primary and secondary analysis. Self-reported data was used, and social desirability may have been a factor impacting reporting. The short dietary questions were used as continuous measures for this analysis, which was on a represented scale, and should not have affected results. The mediation analysis was exploratory, and future upscale of the program will need to adequately plan for analysis investigating different mediation models [48]. Furthermore, this analysis investigated only hypothesised behavioural mediators. Psychological mediators, as explored previously [49, 50], can provide an insights in self-efficacy (confidence) in relation to behavioural changes that have contributed to the weight change.

## Conclusions

TXT2BFiT was effective for both young men and women. Small sustained behavioural changes, including increased vegetable intake and decreased SSB consumption significantly mediated the intervention’s effects on weight change. Improved eating behaviours and increased physical activity accounted for approximately 40 % of the weight change. Thus, our program is suitable for most subgroups and partially explains how the intervention achieved weight loss.

## Abbreviations

BMI: body mass index
CI: confidence interval
IPAQ-SF: International Physical Activity Questionnaire Short Form
MET: metabolic equivalence of task
PA: physical activity
RCT: randomised controlled trial
SES: socioeconomic status
SSB: sugar sweetened beverage
TA: take-away meal
